# New Drugs, Appliances, and Things Medical

**Published:** 1890-08-02

**Authors:** 


### NEW DRUGS, APPLIANCES, AND THINGS MEDICAL.

[All preparations, appliances, novelties, etc., of which a notice is desired, should be sent for The Editor, to care of The Manager, 140, Strand, London, W.C.]

### MEETING OF THE BRITISH MEDICAL ASSOCIATION AT BIRMINGHAM.

Although the exhibits at the meeting of the British Medical Association at Birmingham were perhaps not so numerous as at Leeds last year, there was still a goodly show. In number, and perhaps in completeness, exhibits of surgical instruments take the lead. Among those having stalls we particularly noticed Mayer, of Great Portland Street; Lynch and Co., Aldersgate; Wells and Sons, Oxford Street; Arnold and Sons; Raschke, of Leeds; and Salt, Birmingham. Billington showed excellent wire beds of various designs, and Arnold and Sons various kinds of operating chairs. Ferris had manifold artificial limbs, which would considerably console all losing a natural limb. An invalid lift was also shown by Best and Son, which excited considerable interest. Several opticians were represented, viz., Badly and Curry, and Paxton. Druggists' shows were, of course, strong. Among them we particularly noticed Richardson's soluble pearl-coated pills; the stall of Battley and Wells, chiefly filled with concentrated medical preparations; and the Liverpool Lint Company. Then Johnson and Johnson exhibited numerous kinds of antiseptic dressings; Lang, orthopædic instruments; Schalls, electro-medical apparatus; and Daunton and Co., similar appliances. Jaeger's sanitary clothing was also there in profusion. Leibeg Company's representative fed all who desired with warm "Vivo," manufactured from American meat. Mineral waters were illustrated by the Royal Leamington Spa Company; and medical books by Messrs. Lewis' stall. There was also a collection of photographs of medical celebrities, which have appeared in the *Provincial Medical Journal*. In order to assist strangers during their visit to Birmingham, a specially-prepared guide-book was presented to every member of the British Medical Association; while, as usual, a Daily Journal of Proceedings is issued by the Association.

